# Professors' Expectations About Online Education and Its Relationship With Characteristics of University Entrance and Students' Academic Performance During the COVID-19 Pandemic

**DOI:** 10.3389/fpsyg.2021.642391

**Published:** 2021-04-08

**Authors:** Karla Lobos Peña, Claudio Bustos-Navarrete, Rubia Cobo-Rendón, Carolyn Fernández Branada, Carola Bruna Jofré, Alejandra Maldonado Trapp

**Affiliations:** ^1^Laboratorio de Investigación e Innovación educativa Dirección de Docencia, Universidad de Concepción, Concepción, Chile; ^2^Departamento de Psicología, Facultad de Ciencias Sociales, Universidad de Concepción, Concepción, Chile; ^3^Departamento Currículum e Instrucción, Facultad de Educación, Universidad de Concepción, Concepción, Chile; ^4^Departamento de Bioquímica y Biología Molecular, Facultad de Ciencias Biológicas, Universidad de Concepción, Concepción, Chile

**Keywords:** COVID-19, higher education, online teaching and learning, students experiences, university student

## Abstract

Due to COVID-19, universities have been facing challenges in generating the best possible experience for students with online academic training programs. To analyze professors' expectations about online education and relate them to student academic performance during the COVID-19 pandemic, and considering the socio-demographic, entry, and prior university performance variables of students. A prospective longitudinal design was used to analyze the expectations of 546 professors (54.8% male) in T1. In T2, the impact of the expectations of 382 of these professors (57.6% men) was analyzed, who taught courses during the first semester to a total of 14,838 university students (44.6% men). Professors' expectations and their previous experience of online courses were obtained during T1, and the students' academic information was obtained in T2. A questionnaire examining the Expectations toward Virtual Education in Higher Education for Professors was used. 84.9% of the professors were considered to have moderate to high skills for online courses. Differences in expectations were found according to the professors' training level. The professors' self-efficacy for online education, institutional engagement, and academic planning had the highest scores. The expectations of professors did not directly change the academic performance of students; however, a moderating effect of professor's expectations was identified in the previous student academic performance relationship on their current academic performance.

## Introduction

Due to the COVID-19 pandemic, universities have been facing challenges in creating learning experiences for students using online academic training programs. This new training scenario has tested the adaptability, willingness, and flexibility of faculty members around the world (Quezada et al., 2020). Due to the consequences of the pandemic on teaching and learning processes at all educational levels, there is an urgent need to understand how professors' expectations about online education, are linked to the learning processes and academic performance of their students, considering the changes produced by new forms of teaching and learning.

Theory on the expectations of professors, also known as the Pygmalion effect was presented by Rosenthal and Jacobson (1968), these authors demonstrated that student performance was influenced by teacher expectations. This finding was the beginning of several studies that observed the effect of teacher expectations on the academic performance of their students (Brandmiller et al., 2020). The expectations of professors are defined as the beliefs or assumptions that teachers make about the general levels of behavior and performance of students during their training process (Rubie-Davies et al., 2006). Professor's expectations are explained from a sequence of events such as the existence of stimuli that trigger the teacher's expectations. Then, these expectations are communicated to students and they change, which allows for the generation of behaviors that allow the student to adjust to these expectations impacting student outcomes (Rosenthal, 1994). These beliefs arise from the assessments that professors make based on the characteristics of the subject they teach, about each student, a particular group, or for the course in general (Barriga et al., 2019).

Research on professors' expectations has helped identify how they manage the complexities of the classroom to meet the diverse needs of students (Timmermans et al., 2018). The effects of professor's expectations impact their students through classroom interaction (Hornstra et al., 2018). Therefore, the prior beliefs of educators may influence their motivation to carry out the various instructional activities necessary for the development of the subject matter and affect the academic performance of the students. When professors present negative expectations about their students' performance, they can have a negative influence, especially, in the case of underachieving students (Madon et al., 1997; De Boer et al., 2018).

Professors' expectations about student performance that are systematically too high or too low compared to the students' actual performance level are called biased expectations (Timmermans et al., 2015). Professors may have biased expectations about the whole course or some students (De Boer et al., 2018). In this case, when teachers present diffuse expectations about performance, these may result in a self-fulfilling prophecy; that is, low expectations may hinder student learning, whereas high expectations may foster student learning and eventually lead to higher achievement gains (Gentrup et al., 2020).

A recent systematic review summarizes research published between 1989 and 2018 on the expectations of professors working at different educational levels. The results identify that educators' expectations for their students may be affected by demographic, social-psychological, behavioral, and classroom participation characteristics. However, the authors of this review caution that 30% of the selected studies, in their statistical analyses, did not have student academic performance controlled, making it difficult to establish whether low expectations for a group of students represented a biased professor's expectations or actual reflections based on the students' performance (Wang et al., 2018).

Little research has been conducted in the field of higher education that addresses the issue of professors' expectations in this context (Li and Rubie-Davies, 2016, 2018; Timmermans et al., 2018). Qualitative research that analyzed 20 interviews with university professors reported that both student characteristics (prior academic performance, motivation, and study skills) and professors' characteristics (prior teaching-learning experience and professors' self-efficacy) should be considered influential factors in the formation of professors' expectations (Li and Rubie-Davies, 2018). Therefore, in the process of building professors' expectations, aspects of their students and that of the professors themselves may be involved.

The COVID-19 pandemic has increased anxiety and stress for university students due to the sudden switch from face-to-face teaching to an online learning system. This demands greater autonomy from young students, concentration, and adds concerns about their own physical and mental health, as well as that of their friends and family (Besser et al., 2020; Mseleku, 2020). Because of the pandemic and its consequences, the academic performance of college students has been affected by multiple students, professors, institutional factors, and connectivity-related issues (Adnan and Anwar, 2020; Demuyakor, 2020). For many students, this transition process has been negatively assessed (Garris and Fleck, 2020). A study analyzing the impact of COVID-19 in 30,383 university students in 62 countries indicated that students were primarily concerned about issues related to their future careers and studies, experienced boredom, anxiety, and frustration, and that connectivity difficulty and perceived increased workload prevented them from maintaining and improving their academic performance. Additionally, during the transition process to online education, over 53% of the students were satisfied with the support provided by professors and universities, mainly in Oceania, North America, and Europe (Aristovnik et al., 2020).

The positive evaluation of the students based on the actions of their professors highlights the importance of the relationship between them during this stage. In the COVID-19 scenario, students' perception of belonging and importance is related to high levels of adaptability, and regular opportunities to express their needs and to connect individually with their professors are beneficial for adaptation to this new context (Besser et al., 2020).

Due to the uncertainty of the COVID-19 pandemic, it is of interest to analyze professors' expectations about online education and evaluate its effects on academic performance by considering student characteristics, such as socio-demographic factors, college entrance, and pre-pandemic academic performance. In this regard, analyzing professors' expectations is an important area of research in educational psychology (Wang et al., 2018). This study seeks to provide support from a theoretical and practical point of view. From a theoretical perspective, it will contribute to the literature on the influence of expectations on student academic performance in a specific context of crisis, in addition to clarifying the role of prior academic performance within this relationship. This information will enable us to identify, from a practical point of view, the most relevant expectations that predict student success throughout the semester, which can be useful in implement strategies for the continuous training of professors, in contexts where it is necessary to resort to forced virtual education. This study aimed to analyze professors' expectations about online education and relate them to the academic performance of students during the COVID-19 pandemic, considering the socio-demographic, university entrance, and previous university performance variables of the students.

Therefore, the following assumptions are made: first, we are interested in knowing if (H1) in the face of the COVID-19 pandemic, professors will have positive expectations about online education. Considering the theory of the Pygmalion effect on teacher expectations in university teachers with their students, with the aim examining the possible effects of expectations and their influence on student performance (De Boer et al., 2018). Our second hypothesis seeks to answer whether (H2) professors with previous experience of teaching online courses have higher expectations than inexperienced professors; concerning linking professor expectations to their students. This hypothesis arises from research that posits how different teacher characteristics such as background and beliefs, play a role in the construction of their expectations (Rubie-Davies, 2007; Garcia-Martin and Garcia-Sanchez, 2017; De Boer et al., 2018). In examining the links between teacher expectations and their students, (H3) a positive relationship is expected to be found between professor expectations and student performance during the COVID-19 pandemic, controlling for high school (GPA), college entrance exam (PSU) scores, and the prior career performance of students in higher education. Furthermore, these (H4) differences are expected to be found in the importance of the dimensions of professors' expectations in predicting student performance; and (H5) professors' expectations are expected to moderate the relationship between prior and current student academic performance. All of this is intended to provide data that seeks to answer whether teacher expectations are a true representation based on student performance (Wang et al., 2018).

## Materials and Methods

This study corresponds to a prospective longitudinal design. On a temporal level, it is a longitudinal panel investigation (Ato et al., 2013).

### Participants

During the beginning of the first semester in 2020 (T1), all the professors of a university in the south of Chile were invited to participate in the study. We obtained information about expectations relating to online education during the COVID-19 pandemic from 546 professors (54.8% men and 45.2% women), with an average age of 46.41 years (SD = 11.3). At the end of the academic period (T2), 382 of these professors (57.6% men and 42.4% women) were identified as having taken courses during the first semester for a total of 14,838 university students (44.6% men and 55.4% women; age *M* = 21.67; SD = 2.73) from 95 careers.

Table 1 presents the distribution of students and professors participating in the study, considering the discipline of knowledge of the faculty to which they belong. In general for all disciplines, between 71 and 77% of the professors took courses, except in the Medical and Health Sciences, where only 37% took courses during the first semester, and in the Social Sciences, where it was 90.5%. This difference was significant, X^2^(5) = 97.86, *p* < 0.001.

**Table 1:** Description of participating professors and students.

**Discipline**	**Professors**		**Students**
	**T1**	**T2 (% about T1)(%)**	
Natural Science	109	80 (73.4)	1.131
Agricultural Sciences	71	53 (74.6)	1.911
Medical and Health Sciences	127	47 (37.0)	3.109
Social Science	147	133 (90.5)	5.528
Humanities	39	28 (71.8)	328
Engineering and Technology	53	41 (77.4)	2.831
Total	546	382 (70)	14.838

### Instruments

#### Expectations toward Virtual Education in Higher Education for Professors (CEEVES-D)

The questionnaire on Expectations toward Virtual Education in Higher Education for Professors (CEEVES-D) (Lobos-Peña et al., in preparation) was used to evaluate educators' expectations about online education. As well as measuring expectations about online education; the questionnaire design is self-reporting, and consists of 35 items distributed in nine dimensions, as described in Table 2.

**Table 2:** CEEVES-D dimensions.

**Name**	**Description**	**Cant. of Items**
Institutional Engagement	Refers to the degree of support and resources that the university is expected to provide to the professors	7 ítems
Professors self-efficacy for online education	It shows the capacity to carry out pedagogical, evaluative and administrative processes in a platform	4 ítems
Interaction with students	Defined as expectations to achieve adequate communication and personal relationship with students.	4 ítems
Learning resources and activities	Considers the expected contributions of online activities and resources to the teaching/learning process	4 ítems
Academic planning	Defined as the expectations about communicating and developing the subject according to the planning	4 ítems
Teleworking in the context of crisis	It corresponds to the expectations of generating a space in the home suitable for developing online activities	4 ítems
Comparison with attendance	Defined as the degree to which the online experience will be better or worse than the traditional one in terms of performance, learning and teaching	3 ítems
Online evaluation	It refers to the ability of virtual environments to generate safe assessments that support the teaching/learning process	3 ítems
Monitoring of learning	Related to the ability to follow the learning that the students are doing in the subject	2 ítems

Each item is answered on a five-point Likert scale, where one indicates “strongly disagree” and five indicates “strongly agree.” The higher the score, the higher the professors' expectation of online education is considered high or positive, and scores below three indicate negative or low expectations. Reliability ranged from α = 0.79 to 0.95/ ωt = 0.74 to 0.96 for the dimensions and total scale.

#### Professors' Previous Experiences Teaching Online Courses

The responses were evaluated with oriented questions on the background of the participants' previous use of virtual classrooms, asking about their perception of ability and competence. Three question items were presented: *have you received training in teaching using virtual classrooms? (Virtual Campus, Canvas, other)*, to be answered with two options (Yes/No); as a professor, *have you developed courses using virtual classrooms?* (0, 1, 2, or more), and *how do you evaluate your ability/competence to develop these courses?* (0 = no ability to 3 = high ability).

#### Sociodemographic Characteristics of the Students and the Grade Point Average (GPA)

Gender, university entrance age, high school average grade, the score of university entrance exam (PSU), and type of origin institution were considered as characteristics of the students' university entrance variable. The grade point average (GPA) in the first semester of 2020 and the previous years was obtained from the academic record of the university and considered as the students' academic performance.

### Procedure

This research was endorsed by the Ethics Committee of the participating university, corroborating the ethical criteria for research with human beings. The university entrance data of the students was obtained in March 2020 from the official information registration platforms of the university. The application of the instrument of expectations of professors and their previous experience in online courses was carried out in digital format after obtaining their informed consent, during April 2020, corresponding to the month of the beginning of the academic period, in a virtual format. Finally, the academic performance of the students was obtained at the end of the first academic semester from the LMS CANVAS platform (September 2020).

#### Analysis Plan

All analyses were performed using R version 3.6. Using the information available in the first application, the expectations were analyzed using descriptive statistics. In addition, the relationship between previous experience and the set of expectations was analyzed using a non-parametric multivariate ANOVA test based on 1,000 permutations.

To study the relationship between professors' expectations and student performance in each class, a mixed linear effects model was used, using the information available in T2. As student control variables, gender, age, type of school of origin, and the quadratic effects of high school, entrance scores on the Language Arts and Mathematics test (PSU), and grade point average in the previous semesters in the career since 2015 (university grades) were considered. As control variables of the professors, we used their sex, knowledge discipline, workday, and previous experience in virtual platforms. Regarding the expectations variables, the nine dimensions of the CEEVES-D instrument were considered. To account for the dependence of the data on each other, the Faculty where the student, subject, and professors belonged were considered as random effects. Career was not considered as a random effect, since the variance explained in the different models was very close to 0 and generated estimation errors. To study the difference between various mixed linear models, the likelihood ratio test was used. To evaluate the degree of adjustment between different linear models, the pseudo-R^2^ indicator of Nakagawa and Schielzeth (2013) was used, which allows the evaluation of the level of adjustment to the total variance of the dependent variable as well as the variance explained for each random effect.

## Results

To analyze professors' expectations about online education and relate them to students' academic performance during the COVID-19 pandemic, considering the socio-demographic factors, university entrance, and previous student performance, the results were organized in two sections. First, the results related to university professors' expectations about online education and their relationship with their previous experience were presented; and second, results regarding the link between professors' expectations with the socio-demographic and academic characteristics of their students during the COVID-19 pandemic were presented.

### Professors' Expectations and Previous Experience With Online Education

Table 3 describes the results of the descriptive form of professor's expectations for online education during the COVID-19 pandemic. For all dimensions except the dimension of comparison with attendance, they show averages statistically different from three, which allows us to indicate a positive or negative directionality for each of these. According to the results, it can be affirmed that the dimension of teaching self-efficacy for online education, followed by the dimension of institutional engagement and academic planning, were the dimensions that received the highest score, that is, that professors in these cases present positive expectations about these elements. However, it was observed that the dimension of interaction with students received the lowest score, which was significantly lower than three points (*M* = 2.87).

**Table 3:** Descriptive statistics of CEEVES-D dimensions.

**Dimension**	**Descriptives**				**Test for μ ≠ 3**	
	***M***	**SD**	**Min**	**Max**	***t* (545)**	***p*-value**
Institutional engagement	3.73	0.68	1.43	5.00	25.1	< 0.001
Professors self-efficacy for online education	4.13	0.61	1.00	5.00	43.4	< 0.001
Interaction with students	2.87	0.96	1.00	5.00	3.2	0.001
Learning resources and activities	4.02	0.66	1.00	5.00	36.3	< 0.001
Academic planning	3.84	0.68	1.00	5.00	28.8	< 0.001
Teleworking in the context of crisis	3.75	0.80	1.00	5.00	21.8	< 0.001
Comparison with attendance	2.91	1.12	1.00	5.00	1.9	0.056
Online evaluation	3.39	0.86	1.00	5.00	10.5	< 0.001
Monitoring of learning	3.33	0.95	1.00	5.00	8.04	< 0.001
Total	3.60	0.56	1.26	4.89	25.2	< 0.001

Regarding the results obtained for questions oriented to the previous experience of the professors with online education, it was identified that 93.4% of the professors had received training in teaching an online course. Of the professors, 23.4% had taken an online course and 40% had developed two or more courses. On the self-evaluation for taking online courses, 84.9% considered that they had moderate to high skills for taking online courses. Using a non-parametric multivariate ANOVA test with 1,000 permutations, only significant differences were found in expectations attributable to participation in training courses (see Table 4), *F*(3.6, 473.4) = 2.7, pperm = 0.039, but not with respect to the course completion, *F*(6.8, 1746.9) = 0.618, pperm = 0.758, nor the perception of ability, *F*(4.82, 71.47) = 0.852, pperm = 0.478. The group that received training presents higher values on all scales, with differences ranging from weak to moderate when considering effect sizes. Only the difference between people with and without training in online evaluation was significant, *p* = 0.003, *d* = 0.54.

**Table 4:** Differences in expectations between people with and without training in virtual education.

	**Without training** ** (*n* = 36)**		**With training** ** (*n* = 510)**				
**Dimension**	***M***	**SD**	**M**	**SD**	***t***	***p*-value**	**d**
Institutional engagement	3.62	0.70	3.74	0.68	*t*(39.8) = 0.97	0.337	0.17
Professors self-efficacy for online education	4.03	0.73	4.13	0.60	*t*(38.3) = 0.85	0.400	0.18
Interaction with students	2.78	1.02	2.87	0.96	*t*(39.5) = 0.51	0.614	0.09
Learning resources and activities	3.75	0.86	4.04	0.64	*t*(37.7) = 2.00	0.053	0.45
Academic planning	3.70	0.72	3.85	0.68	*t*(39.5) = 1.17	0.250	0.21
Teleworking in the context of crisis	3.51	0.84	3.76	0.80	*t*(39.6) = 1.74	0.090	0.31
Comparison with attendance	2.65	0.95	2.93	1.13	*t*(42.3) = 1.68	0.100	0.25
Online evaluation	2.95	0.84	3.42	0.85	*t*(40.2) = 3.19	0.003	0.54
Monitoring of learning	3.08	1.02	3.34	0.94	*t*(39.3) = 1.49	0.145	0.27
Total	3.41	0.61	3.62	0.55	*t*(39.2) = 1.95	0.058	0.37

### Teaching Expectations and Their Link to Student Academic Performance

Linear mixed models were used to model the relationship between professors' expectations and student performance (H3). In the initial model investigation, non-linear relationships between students' previous and current performance were found; hence, they were modeled as quadratic effects. Specifically, the difference between the three models was tested: Model 1, with control variables for professors and students; Model 2, which considers the effect of expectations on performance; and Model 3, which considers interaction effects between previous performance and professor's expectations on performance during 2020.

Using the likelihood ratio test, no significant differences were found between Models 1 and 2, X^2^ (9) = 8.82, *p* = 0.45, indicating that professors' expectations have no direct effect on students' current academic performance. However, there are significant differences between Models 2 and 3, X^2^ (81) = 213,506, *p* < 0.001, which indicates that teaching expectations influence performance, but as mediators of the effect of previous performance on current performance.

Table 5 shows the parameters and adjustment indicators for Models 1 and 2; the parameters of Model 3 are available as supplementary information (see Supplementary Material). In both Models 1 and 2, the coefficients for student gender, type of educational institution of origin, GPA, PSU Language and Mathematics, previous university grade, and professor discipline are significant. When analyzing the adjustment indicators of Model 3, the pseudo-R^2^ goes from 0.145 in Model 2 to 0.153 in Model 3. The level that explains this improvement is the level 1—of grades in each subject, whose pseudo-R^2^ goes from 0.003 in Models 1 and 2 to 0.01 in Model 3.

**Table 5:** Model 1, 2 and 3 adjustment coefficients and indicators.

	**Model 1**		**Model 2**	
**Coefficient**	**Estimate**	**valor-*p***	**Estimate**	**valor-*p***
**Fixed effects** (Intercept)	−0.59	0.152	−0.53	0.207
Student gender = Male	−0.10	< 0.001	−0.10	< 0.001
Age at entry	0.00	0.771	0.00	0.780
Type of establishment = Private paid	0.07	< 0.001	0.07	< 0.001
Type of establishment = Particularly subsidized	0.04	< 0.001	0.04	< 0.001
Establishment type = No information	0.01	0.808	0.01	0.805
Secondary Notes	0.10	< 0.001	0.10	< 0.001
Secondary notes^2^.	0.01	< 0.001	0.01	< 0.001
PSU Language	0.03	< 0.001	0.03	< 0.001
PSU Language^2^	−0.01	0.003	−0.01	0.003
PSU Math	0.04	< 0.001	0.04	< 0.001
PSU Mathematics^2^	0.01	0.026	0.01	0.026
No previous university grade	0.65	0.004	0.65	0.004
Previous notes university	0.62	< 0.001	0.62	< 0.001
Previous university grades^2^.	0.32	< 0.001	0.32	< 0.001
Professors gender = Male	−0.09	0.204	−0.09	0.211
Teaching discipline = Agricultural Sciences	0.48	< 0.001	0.48	< 0.001
Teaching discipline = Medical and health sciences	0.52	< 0.001	0.56	< 0.001
Professors.discipline=Social Sciences	0.27	0.009	0.27	0.009
Teaching discipline = Humanities	0.21	0.163	0.23	0.123
Teaching discipline = Engineering and technology	0.24	0.076	0.25	0.072
Teaching day = more than 22 h	0.02	0.840	0.01	0.860
Experience–Training: Yes	0.09	0.516	0.07	0.630
Experience–Courses : 1	−0.04	0.652	−0.05	0.583
Experience–Courses : 2 or more	0.02	0.820	0.03	0.732
Self-reported ability: low skill	−0.21	0.603	−0.26	0.521
Self-reported ability: moderate ability	−0.12	0.757	−0.18	0.649
Self-reported ability: highly skilled	0.01	0.985	−0.06	0.890
Institutional engagement			−0.01	0.799
Professors self-efficacy for online education			0.12	0.013
Interaction with the student			0.02	0.687
Learning resources and activities			−0.02	0.626
Academic planning			−0.02	0.626
Teleworking in the context of crisis			0.00	0.959
Comparison with attendance			0.01	0.800
Online evaluation			0.03	0.617
Monitoring of learning			−0.05	0.329
**Random effects**
σ Student	0.296		0.296	
σ Subject	0.497		0.496	
σ Professors	0.515		0.519	
σ Faculty	0.079		0.068	
σ Residual	0.532		0.532	
**pseudo-*****R***^2^ Nakagawa and Schielzeth (2013)
General	0.138		0.145	
Subject	0.410		0.410	
Faculty	0.077		0.080	
Student	0.023		0.005	
Professors	0.870		0.903	
Level 1–Subject	0.003		0.003	

*It is considered as a reference point the female gender of professors and student, municipal establishment, discipline of the Natural Sciences professors, teaching day less than 22 h, no training in virtual teaching, no courses taken and no self-informed skills for online teaching*.

**Figure 1:**
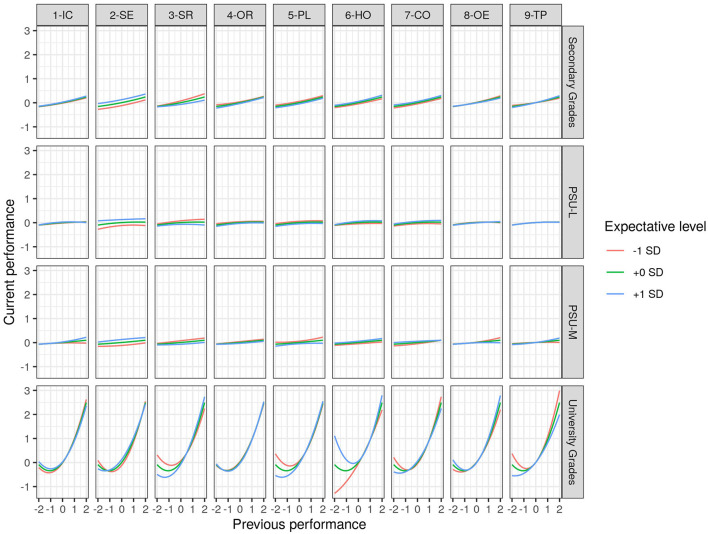
Moderation of expectations in the relationship between previous and current performance.

Figure 1 shows the current performance prediction curves as a function of previous performance, moderated by each of the expectation dimensions. In general, the variable that has the strongest level of influence on current performance is the previous performance in the subject. This relationship is U-shaped, with the lowest point located in note 5.0, being the minimum considered sufficient. The rest of the variables (GPA, PSU Language, and PSU Math) present approximately direct and linear relationships, although of less intensity.

When analyzing the patterns by expectation variables, the professor's self-efficacy dimension for online education shows a pattern of parallel curves, where the form of the relationship is maintained for all levels of self-efficacy. However, the averages are higher for professors with greater self-efficacy. In the variable where the moderating effect is most strongly observed, it is in the previous performance, specifically in the grades under the inflection point of the U-curve. Two patterns stand out. First, for the variables of student interaction, academic planning, and monitoring learning, performance for students under the tipping point is lower if professors have high expectations for these variables. Concerning the second pattern, in professors who have low expectations for teleworking in a crisis context, the U pattern is broken and a linear relationship is observed between previous performance in the university and current performance, which does not occur in professors with medium or high expectations, where the usual U pattern is replicated.

The relative effect of each of the dimensions is analyzed, by considering the difference between the complete Model 3 and Model 3 by eliminating each of the dimensions separately. In Table 6, it can be observed that six of the dimensions (Institutional Engagement, Interaction with the student, Academic Planning, Comparison with the attendance, Evaluation in line, and Monitoring to the learning) when being eliminated from the complete Model 3, generate significant differences in the predictive capacity of the model.

**Table 6:** Effect of each expectation dimension on Model 3.

**Dimension**	***X*^2^(10)**	***p*-value**
Institutional engagement	23.4	0.009
Professors self-efficacy for online education	16.52	0.086
Interaction with students	41.5	< 0.001
Learning resources and activities	6.24	0.795
Academic planning	22.84	0.011
Teleworking in the context of crisis	17.90	0.057
Comparison with attendance	33.91	< 0.001
Online Evaluation	25.65	0.004
Monitoring of learning	31.76	< 0.001

*Likelihood ratio test compares Model 3, against Model 3 without each specific dimension*.

## Discussion

This study aimed to analyze professors' expectations about online education and relate them to students' academic performance during the COVID-19 pandemic, considering sociodemographic factors, university entrance, and the previous university performance of the students. The professors' expectations for online education in the COVID-19 pandemic and student performance are discussed in relation to their previous experiences with virtual education.

### Professors' Expectations and Previous Experience With Virtual Education

The results identified generally positive expectations for online education in educators during the COVID-19 pandemic. H1 was confirmed (professors had positive expectations for online teaching). Positive expectations were identified in the professors' self-efficacy for online education, that is, professors' belief in their ability to teach online. This is followed by the dimension of institutional engagement, which evaluates the university's ability to provide technological and pedagogical support to address delivery of subjects and academic planning, a dimension referred to the expectations in communicating and developing the subject according to the planning.

Professors' self-efficacy for online education is defined as judgments about their ability to achieve the desired results in student learning and participation. These beliefs affect the effort that professors invest in the teaching process, which benefits planning and organization, applying new teaching methodologies, and meeting students' needs (Tschannen-Moran and Woolfolk Hoy, 2001). Studies report that professors are more confident in carrying out their professional work (Giménez-Lozano and Morales-Rodríguez, 2019). Professors' self-efficacy is considered as an indicator that can make a difference in the learning outcomes of students in their course (Hampton et al., 2020).

Regarding institutional engagement, professors feel confident about the contribution of the university as an institution in the process of transitioning to online education due to COVID-19. These results are congruent with a meta-analysis study describing the different typologies adopted by universities around the world during the pandemic. Many universities have shown commitment in the transition process to online education due to the pandemic, and institutions have taken advantage of the potential created by forced virtualization to facilitate flexible and innovative digital education methods (Crawford et al., 2020), facilitating the construction of platforms and resources linked to quality online education.

The third dimension, with positive expectations from professors, was academic planning, which referred to the expectations in communicating and developing the subject according to planning. For an appropriate development of teaching in online environments, professors need to be prepared and motivated to re-design instruction with good pedagogical sense and effectiveness. Therefore, professors feel expectations to modify and adapt their traditional planning models to new procedures and methodologies that contribute to improving the quality of teaching and learning processes at distance. Authors, such as Green et al. (2020), report that in other countries, the transition process due to the pandemic began during the first semester of 2020; at that time, professors in different countries had to quickly redesign what they had prepared in advance for the semester, unlike professors in Chile, who began the academic year during March 2020 and were able to evaluate the scenarios adopted in other institutions at the beginning of the year.

Low expectations were identified in terms of student interaction. This finding is similar to that reported in other studies, where university professors feel less competent in the relationships they establish with their students during the development of online courses (Hampton et al., 2020); although professors have positive expectations regarding academic issues, it seems that the establishment of social relationships with their students is a process that is weakened. These beliefs on the part of the professors may be a consequence of the evaluation processes that professors carry out, based on the characteristics of the subject they teach and on their students (Barriga et al., 2019). However, it is important to note that the relationships between students and their professors are associated with academic results, participation, and the risk of dropout by young people (Kincade et al., 2020).

Regarding the second hypothesis, which refers to the effects of previous experiences teaching online courses on professors' expectations (H2), we found an effect of training in virtual education, where untrained professors had lower expectations than trained ones. It is important to consider this finding during the transition to online education in the COVID-19 pandemic, and training educators in online learning environments contributes to the quality of teaching planning and facilitation (Amador Solano and Espinoza Guzmán, 2017). Training provides professors with the opportunity to strengthen their skills and knowledge to drive learning within virtual environments (Odunaike et al., 2013).

### Teaching Expectations and Their Link to Student Academic Performance

With respect to linking professor expectations to their students, (H3), the results did not identify relationships between professors' expectations and their students' current academic performance, after controlling for the previous grade (high school GPA), scores on university selection tests (PSU), and previous performance (GPA) in the career of higher education students. It seems that the student's academic performance is a variable that is more related to elements of the student, such as motivational factors like academic self-efficacy, self-regulation in learning, and effort regulation, etc. (Richardson et al., 2012). Likewise, in H4, where it was expected to find differences in the importance of the dimensions of teaching expectations in the prediction of student performance, it was possible to identify how the different dimensions of the questionnaire on teaching expectations independently moderate the relationship between the previous academic performance of students and current academic performance. This finding is related to the results presented by Adnan and Anwar (2020), who argue that, due to the pandemic and its consequences, the academic performance of college students has been affected by multiple factors, including educator-related factors.

Finally, in response to H5, which hypothesized that expectations moderate the relationship between previous and current educational performance. From the results, it was possible to find a statistically significant moderating effect of professors' expectations on changes in the relationship between students' previous academic performance and current academic performance. This finding is important, particularly because it appears that the greatest influence is found in how expectations affect previously underperforming college students, strengthening or decreasing the inverse relationship between previous and current pandemic performance in that group.

Although a limitation of the longitudinal research is the loss of participants in T2. The participants belonged to a single university, which, although it includes students from all over the country, these results should be incorporated or analyzed in teachers from other universities, both public and private. Another limitation could be that the questionnaire was measured in the context of emergency education, which may alter the findings with respect to the factorial structure of the instrument and its subsequent analysis. Further research on the subject is suggested.

Among the strengths of this study, is its contribution regarding educational expectations in the context of higher education and their relationship to the success of university students, a line of research where few studies have been identified (Li and Rubie-Davies, 2016, 2018; Timmermans et al., 2018). It has also presented results on the effect of educator expectations on the academic performance of their students, statistically controlled for other variables of interest, such as previous academic performance, which is particularly valuable in noting the strength of the relationship between previous university performance and current performance (Wang et al., 2018).

The practical implications of these findings lie in the importance of professors' expectations regarding online education and its effects on changing student academic performance, especially during the first academic year where young people are in a process of adapting to the new demands of university life (Cobo-Rendón et al., 2020). For universities, these results are useful in the construction of strategies for continuous professor training, where it is possible to promote positive expectations in the development of online courses and positively impact educators and students.

Future studies could investigate how professors' expectations serve as moderators in other cognitive variables that motivate students, such as academic self-efficacy, academic engagement, and self-regulation of learning, which are essential for adaptation to and success in university.

Studies of this type evaluate interested professors and improve their performance by identifying areas of virtual education with lower expectations. It also allows professors to identify “strengths” in virtual education that they could then maintain and enhance. It is beneficial to anticipate expectations and identify the dimensions that contribute to university decision-making in educational policy and the impact of expectations on critical careers. For universities, these results are useful in the construction of strategies for continuing teacher education, where it is possible to promote positive expectations in the development of online courses, and positively impact both professors and students.

## Data Availability Statement

The original contributions presented in the study are included in the article/Supplementary Material, further inquiries can be directed to the corresponding author/s.

## Ethics Statement

The studies involving human participants were reviewed and approved by the ethics committee of university of Concepcion. The patients/participants provided their written informed consent to participate in this study.

## Author Contributions

KL contributed to the design of the study, literature, and writing of the manuscript. CB-N contributed to the design of the study as well as data extraction, data analysis, and review of the abstract and manuscript. RC-R and CF contributed to the design of the study, interpretation of the results, and review of the abstract and manuscript. CB and AM contributed to the interpretation of the results and writing the manuscript. All authors contributed to the article and approved the submitted version.

## Conflict of Interest

The authors declare that the research was conducted in the absence of any commercial or financial relationships that could be construed as a potential conflict of interest.
